# Pressure-Induced Stabilization of Terbium(IV) in CsTb(CrO_4_)_2_ Characterized by X‑ray Absorption Spectroscopy

**DOI:** 10.1021/jacs.6c06234

**Published:** 2026-06-16

**Authors:** Tyler W. Hines, Lucia Amidani, Nicholas B. Beck, Kacy N. Mendoza, Christoph Sahle, Joseph M. Sperling, Sami Vasala, Kristina O. Kvashnina, Thomas E. Albrecht

**Affiliations:** † Department of Chemistry and Nuclear Science & Engineering Center, 3557 Colorado School of Mines, Golden, Colorado 80401, United States; ‡ The Rossendorf Beamline, 55553 European Synchrotron Radiation Facility, 71, Avenue des Martyrs, CS 40220, Grenoble Cedex 9 38043, France; § Helmholtz-Zentrum Dresden-Rossendorf, Institute of Resource Ecology, Bautzner Landstraße 400, Dresden 01328, Germany; ∥ European Synchrotron Radiation Facility, 71 Avenue des Martyrs, Grenoble 38043, France

## Abstract

Single crystals of CsTb­(CrO_4_)_2_ and CsDy­(CrO_4_)_2_, where the lanthanide metals are in the trivalent state under ambient conditions, have been investigated under high-pressure conditions on the gigapascal scale utilizing a diamond anvil cell. These compounds were characterized by single-crystal X-ray diffraction in addition to high-pressure solid-state UV-vis-NIR spectroscopy, Raman spectroscopy, and Tb L_3_-edge high-energy-resolution fluorescence-detected X-ray absorption near-edge structure (HERFD-XANES). The high-pressure UV-vis-NIR spectra reveal strong broadening of the metal-to-ligand charge transfer band to lower energies, associated with a visible color change from yellow to dark red/black. Clear evidence for the stabilization of Tb^4+^ under high pressure is provided by the appearance of a second edge feature characteristic of Tb^4+^, starting at 19.62 GPa in the high-pressure L_3_-edge HERFD-XANES at around 7528 eV. This represents the first example of Tb^4+^ being stabilized by high pressure and expands upon the limited chemistry of terbium in the tetravalent state.

## Graphical abstract

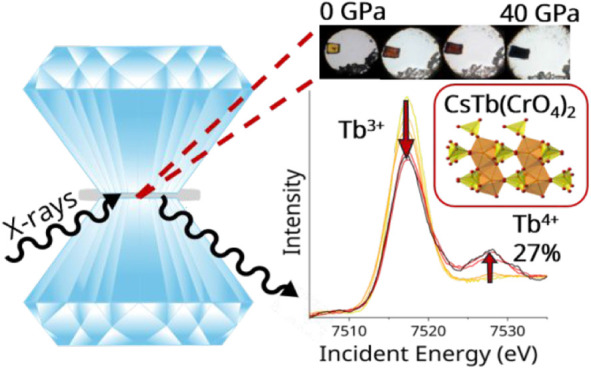

## Introduction

The chemistry of the lanthanide elements is dominated by the most stable 3+ oxidation state across the series, owing to the core-like behavior of the valence 4*f* electrons. However, some lanthanides can be stabilized in the divalent or tetravalent state, given appropriate ligands and coordination geometry.^1–4^ Of these, the divalent lanthanides are more widely used, mostly as strong, one-electron reductants in organic synthesis, as exemplified by the use of SmI_2_ and its derivatives.^2^ Additionally, stabilization of divalent lanthanides is generally much more achievable, with examples of every lanthanide (except radioactive promethium) isolated in the divalent state.^2–5^ Of the lanthanides, the divalent oxidation state is most common for Eu and Yb, stemming from their divalent electronic configuration, which results in a half-filled 4*f* ^7^ (Eu^2+^) or filled 4*f* ^14^ (Yb^2+^) subshell.^2,5^

Similarly, although the tetravalent state in the lanthanides is more elusive, the stabilization provided by the 4*f* ^0^ and 4*f* ^7^ electron configurations in cerium and terbium provides Ln^3+/4+^ oxidation potentials of −1.6 V and −3.1 V, respectively, resulting in molecules and materials that can be isolated, albeit with difficulty for Tb^4+^.^1^ Ce is by far the most common due to the much lower Ce^3+/4+^ oxidation potential of −1.6 V, with examples of molecular complexes stabilized under atmospheric conditions, such as the common Ce^4+^ starting material, ceric ammonium nitrate. Contrasting this with Tb^4+^ and Pr^4+^, the difficulty of stabilizing these two states is obvious, given the limited number of molecular complexes isolated in the solid state and the stringent anaerobic conditions and carefully designed ligands necessary to stabilize them.^6–11^ This does beg the question, however, of whether the physical compression of a hard donor ligand–metal ion bond can stabilize higher oxidation states, as described by Pearson’s Hard Soft Acid Base (HSAB) Theory.^12^

Pressure-induced valence transitions in lanthanide compounds are known; however, they are largely limited to the divalent-to-trivalent transition of the metals, monochalcogenides, and intermetallic compounds of europium, samarium, thulium, and ytterbium that are readily accessible in their +2 states.^13–31^ Ce is the only lanthanide element with literature precedent to undergo a pressure-induced valence fluctuation from trivalent to tetravalent in the metallic phase, in addition to some chalcogenide, pnictide, and intermetallic compounds.^30,32–34^

Outside of the *f* elements, pressure-induced valence changes have been demonstrated in examples that include FeTiO_3_, TlReO_4_, CuFeO_2_, BiNiO_3_, and PbCrO_3_.^31,35–38^ Chromium specifically exhibits a wide variety of stable oxidation states from −2 to +6, leading to rich redox chemistry, as evident by the pressure-induced stabilization of Cr^4+^ at 4.2 GPa in PbCrO_3_.^31^ For the *f* block elements specifically, Cs_2_[(UO_2_)_2_(CrO_4_)_3_] and Dy­(CrO_4_) compounds have been synthesized, stabilizing chromium in the rare Cr^5+^ oxidation state that commonly undergoes disproportionation.^39,40^ Additionally, the compound CsAm­(CrO_4_)_2_ with chromium in its common +6 state, exhibits a broad, low-energy 5*f →* 6*d* metal-to-ligand charge-transfer (MLCT) band that arises from the accessible Am^4+^ state (Am^3+/4+^ = −2.62 V vs S.H.E).^41^ The low-energy MLCT band in CsAm­(CrO_4_)_2_, stabilization of Cr^5+^ in Ln­(CrO_4_), and pressure-induced charge transfer through bridging O^2–^ anions in similar systems suggest that trivalent rare earth compounds with chromate anions in their common +6 oxidation state may facilitate the stabilization of lanthanides in the tetravalent state with the application of pressure.^31,35–41^

Herein, the possibility of pressure-induced oxidation of lanthanides is investigated in CsLn­(CrO_4_)_2_ (Ln^3+^ = Tb^3+^ and Dy^3+^). These compounds have been characterized using high-pressure UV-vis-NIR and Raman spectroscopies, in addition to high-pressure Tb L_3_-edge high-energy-resolution fluorescence-detected X-ray absorption near-edge structure (HERFD-XANES) for CsTb­(CrO_4_)_2_. Utilizing these methods, we report evidence of the pressure-induced stabilization of Tb^4+^ in CsTb­(CrO_4_)_2_.

## Experimental Section

### Materials

Cs_2_CrO_4_ (99.5%, Alfa Aesar), HNO_3_ (ACS reagent, 70%, Sigma-Aldrich), Tb_4_O_7_ (99.9%, Sigma-Aldrich), and Dy_2_O_3_ (99.9%, Sigma-Aldrich) were used as received. Ln­(NO_3_)_3_·nH_2_O (Ln^3+^ = Tb^3+^, Dy^3+^) were synthesized by dissolving the respective lanthanide oxide in concentrated HNO_3_, followed by drying to completeness resulting in crystalline Ln­(NO_3_)_3_·nH_2_O. These nitrates were assumed to be in the hexahydrated Ln­(NO_3_)_3_·6H_2_O form.

### Syntheses

CsLn­(CrO_4_)_2_ (Ln = Tb, Dy) were synthesized in a hydrothermal reaction based on previous methods by Galley et al.^41^ The synthesis of CsCe­(CrO_4_)_2_ was attempted, but the addition of Cs_2_CrO_4_ to Ce­(NO_3_)_3_·6H_2_O resulted in the immediate oxidation of cerium and produced brown amorphous powder products. In a typical synthesis, 0.2 mmol Ln­(NO_3_)_3_·6H_2_O, 0.4 mmol Cs_2_CrO_4_, and 2 mL H_2_O were loaded into a 23 or 11 mL PTFE-lined autoclave. The autoclaves were sealed and heated at 200 °C for 48 h in a box furnace and allowed to cool to room temperature over 48 h (cooling rate ∼3.7 °C/h). The resulting product was filtered, washed with deionized water to remove excess Cs_2_CrO_4_, and dried. This yielded well-faceted orange or yellow single crystals with a block crystal habit for CsTb­(CrO_4_)_2_ and CsDy­(CrO_4_)_2_, respectively. The bulk material can be viewed in the Supporting Information (Figure S1). Yields for these syntheses were greater than 54% (Table S1).

### Crystallographic Studies

Single crystals of CsTb­(CrO_4_)_2_ and CsDy­(CrO_4_)_2_ were submerged in immersion oil and placed on a 75 μm diameter MiTeGen loop. The loops with CsTb­(CrO_4_)_2_ and CsDy­(CrO_4_)_2_ were mounted on the goniometer of a Bruker D8 Quest diffractometer and aligned with the incident X-ray beam using a digital camera integrated with the ApexV software. Collection strategies were calculated and optimized using the ApexV software. The data were collected with Mo K_α_ (λ = 0.71093 Å) radiation source at 100 K for CsTb­(CrO_4_)_2_ and 150 K for CsDy­(CrO_4_)_2_ using an Oxford 1000 cryostream. The CsTb­(CrO_4_)_2_ structure was refined on the Olex2 v1.5 software using the SHELXT structural solution and SHELXL structural refinement.^42–44^ The CsDy­(CrO_4_)_2_ structure was refined on the Olex2-v1.5 software using the SHELXS structural solution and SHELXL structural refinement.^42–44^ Powder X-ray diffraction was collected on a Bruker D2 Phaser between 2θ angles of 5° and 50°. Powder patterns can be found in the Supporting Information (Figures S3–S4).

### Ambient Pressure Solid-State UV-vis-NIR Spectroscopy

Single crystals of CsTb­(CrO_4_)_2_ and CsDy­(CrO_4_)_2_ were placed on a glass slide and submerged in immersion oil. The solid-state UV-vis-NIR transmission data for these samples were collected on a CRAIC Technologies microspectrometer at room temperature. Data were collected between 320 and 1700 nm, and collection times were optimized by the CRAIC Minerva software.

### Ambient Pressure Solid-State Raman Spectroscopy

Single crystals of CsTb­(CrO_4_)_2_ and CsDy­(CrO_4_)_2_ were placed on a glass slide. The solid-state Raman spectra were collected on a Bruker Senterra II Raman microscope by using a 785 nm laser at a power of 100 mW, with 20 coadditions and an integration time of 1000 ms. Raman spectra were baseline-corrected in the OPUS 8.7.41 software.

### Ambient Pressure Solid-State X-ray Absorption Spectroscopy

HERFD-XANES at the Tb L_3_-edge was acquired on BM20 at the ESRF synchrotron facility.^45^ The incident energy was selected with a fixed-exit Si(111) double-crystal monochromator. A Rh-coated toroidal mirror was used to focus the beam to 2.5 × 0.075 mm on the sample. The horizontal size of the beam was reduced to 0.5 mm by using slits. HERFD-XANES was acquired by integrating a small bandwidth around the maximum of the Lα_1_ emission of Tb while scanning the incident energy across the Tb L_3_ absorption edge. The maximum of the Tb Lα_1_ emission line was selected by the fourth harmonic of 4 Ge(110) bent crystal analyzers mounted on a Johann-type X-ray emission spectrometer and working at a Bragg angle of 81.18°.^46^ The spectrometer was set to a 0.25 m Rowland radius and equipped with crystal analyzers bent at a 0.5 m radius to maximize the solid angle covered by the spectrometer. Data were processed using PyMCA software, including the normalization and background subtraction tools integrated therein. Quantification analysis was performed using ITFA software.

Single crystals of CsTb­(CrO_4_)_2_ were placed on a Kapton tape and mounted on the sample stage to collect ambient-pressure HERFD-XANES.

### Variable Pressure UV-vis-NIR, Raman, and X-ray Absorption Spectroscopy

For the high-pressure UV-vis-NIR and Raman spectroscopy experiments, a 200 μm diameter hole was drilled into a stainless-steel gasket, indented to 100 μm, and mounted onto a DACTools (Naperville, IL, USA) SSDAC 80 diamond anvil cell (DAC). Single crystals of CsTb­(CrO_4_)_2_ and CsDy­(CrO_4_)_2_ were loaded into the mounted stainless-steel gasket along with ruby spheres as a pressure reference.^47–50^ The gasket hole was filled with poly­(dimethylsiloxane) as the pressure medium to ensure hydrostatic transfer of pressure throughout the cell, and the cell was sealed. The ruby fluorescence was measured on a CRAIC Technologies microspectrometer with an excitation wavelength of 546 nm to determine the pressure inside the DAC at each pressure step.^47,50^ Ruby fluorescence was fit to a Voigt function to determine the λ_max_. For the high-pressure X-ray absorption spectroscopy experiments, a 200 μm diameter hole was drilled into a beryllium gasket, indented to 100 μm, and mounted onto an mBX 110 diamond anvil cell.^51^ Single crystals of CsTb­(CrO_4_)_2_ were loaded into the mounted beryllium gasket along with ruby spheres as a pressure reference.^47–50^ The gasket hole was filled with a 4:1 methanol:ethanol mixture as the pressure medium, and the cell was sealed. HERFD-XANES was not collected for CsDy­(CrO_4_)_2_. The ruby fluorescence was measured on a BETSA Pressure by Ruby Luminescence System at each pressure step. Ruby fluorescence was fit to a Voigt function to determine the λ_max_. Solid-state UV-vis-NIR, Raman, and X-ray absorption spectra were then collected at various pressures in the loaded DAC based on the methods above.

## Results and Discussion

Both CsTb­(CrO_4_)_2_ and CsDy­(CrO_4_)_2_ crystallize in the monoclinic space group *P*2/*c*, isostructural to previously reported lanthanide chromates, with one crystallographically unique Ln^3+^ cation, Cs^+^ cation, and CrO_4_^2–^ anion in the asymmetric unit.^41,52^ This layered structure is visualized in Figure 1(a) viewed along the *c*-axis. A picture of the asymmetric unit with thermal ellipsoid plots can be found in the Supporting Information (Figure S2). The CrO_4_^2–^ tetrahedra share corners and edges with the Ln^3+^ polyhedra to form layers, and these layers are separated by Cs^+^ cations filling the interlayer space. The Ln^3+^ and Cs^+^ cations sit on 2*e* (0, *y*, 1/4) and 2*f* (1/2, *y*, 1/4) Wyckoff positions, respectively, with 2-fold site symmetry. The Cr^6+^ cations sit on the 4*g* (*x*, *y*, *z*) general Wyckoff position.^53^ The local geometry (Figure 1(b)) of the Ln^3+^ polyhedra and CrO_4_^2–^ units is triangular dodecahedron with *D*_2*d*_ symmetry and tetrahedral with *T*_*d*_ symmetry, respectively. Bond lengths, unit cells, and other crystallographic information are similar between the two structures, following the lanthanide contraction, and can be found in the Supporting Information (Tables S2–S3). Powder patterns can also be found in the Supporting Information (Figures S3–S4).

**Figure 1:**
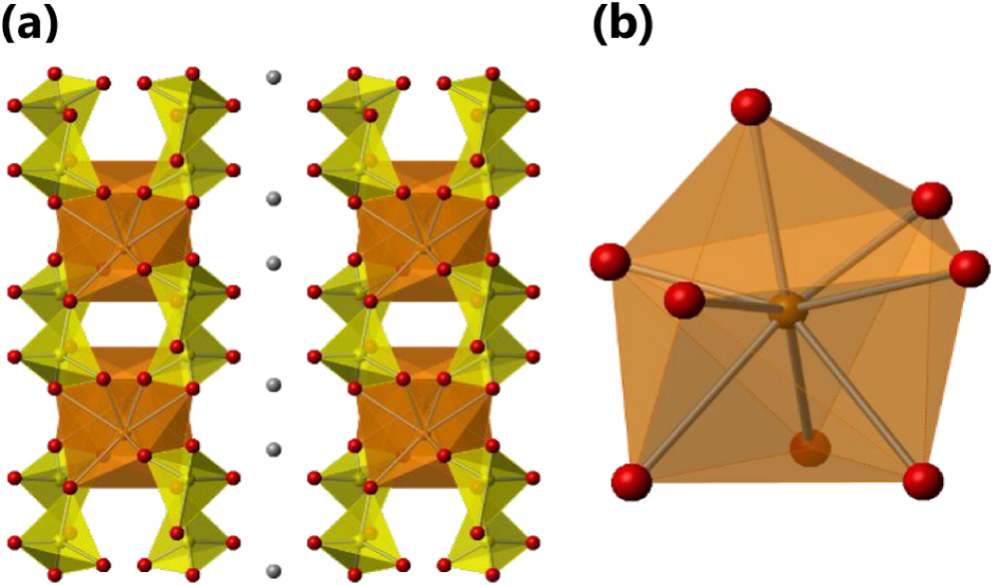
Polyhedral representations for CsLn­(CrO_4_)_2_. (a) Depicts the extended structure viewed along the *c*-axis to visualize the layered structure, and (b) the eight-coordinate trigonal dodecahedral local geometry of the Ln^3+^ centers. Terbium/dysprosium, chromium, oxygen, and cesium atoms are represented by orange, yellow, red, and gray spheres, respectively. Tb^3+^/Dy^3+^ and CrO_4_^2–^ are represented by orange and yellow polyhedra, respectively.

Tracking the Laporte Forbidden *f→f* transitions of the *f* elements can provide insights into the underlying electronic structure and *f* electron perturbations as a function of pressure.^54–57^ Unfortunately, Tb^3+^ 4*f*→4*f* transitions have the lowest probabilities in the lanthanide series. Additionally, the most intense transitions for Tb^3+^ are in the region of 350 to 400 nm and are obscured by the ligand-to-metal charge-transfer (LMCT) of the chromate oxoanion. The same is true for Dy^3+^ with the exception of the ^6^H_15/2_→^6^F_5/2_ transition at about 800 nm that is barely observed at ambient pressure to *ca*. 6 GPa (Figure S6) even though the molar absorptivity of this transition is about five times larger than the most intense Tb^3+^ peak (Figure 2b).^58^ At ambient pressure, without being in a DAC, several more 4*f*→4*f* transitions are observed (Figure S5). Thus, the 4*f*→4*f* transitions of Tb^3+^ and Dy^3+^ cannot be utilized to analyze the electronic structure of the lanthanide metal center as they have been in other recent high-pressure spectroscopic studies of the *f* elements.^54,55,57,59,60^ Despite this, the charge-transfer and 4*f*→5*d* bands can still provide valuable information.

**Figure 2:**
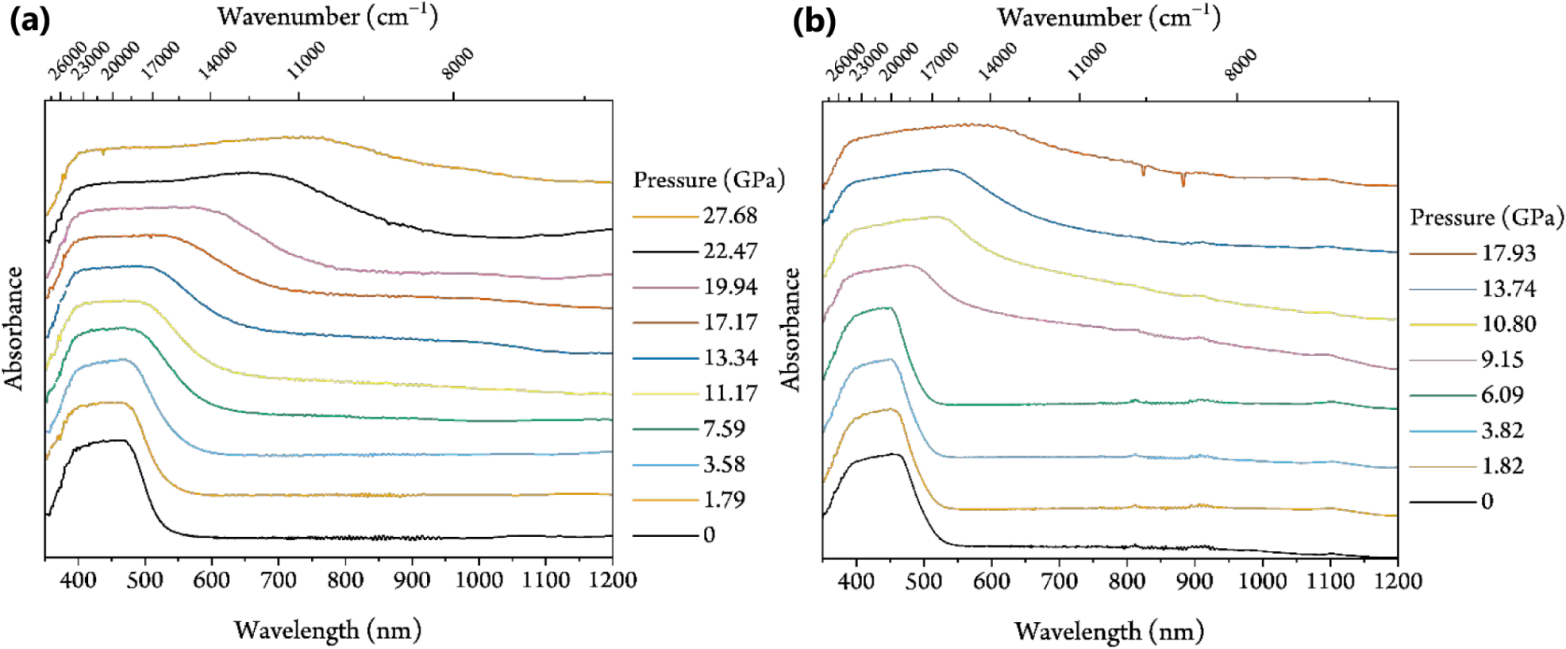
High-pressure UV-vis-NIR absorption spectra of (a) CsTb­(CrO_4_)_2_ and (b) CsDy­(CrO_4_)_2_.

At ambient pressure, the solid-state UV-vis data reveal that CsTb­(CrO_4_)_2_ has a lower energy feature than that of CsDy­(CrO_4_)_2_ corroborating the darker orange color of the bulk CsTb­(CrO_4_)_2_ crystals (Figure S5). This lower energy feature is almost certainly a combination of 4*f*→5*d* and MLCT transitions, given that the Tb^3+/4+^ oxidation potential is lower than Dy^3+/4+^ and is the second most feasible in the lanthanide series, where even molecules with Tb^4+^ have now been synthesized.^1,6–8,10,11^ It has been known for over half a century that there is a linear correlation between these transitions and the ion’s redox behavior.^61,62^ Additionally, the oxidizing power of CrO_4_^2–^ is enough to easily oxidize Ce^3+^.

As observed in Figure 2a, the MLCT band in CsTb­(CrO_4_)_2_ is clearly broadened to include lower energies with the application of pressure and is associated with a visual color change from yellow-orange to dark red/black, as observed in Figure 3(a). This suggests that the charge transfer from the Tb^3+^ cations to the CrO_4_^2–^ anions is becoming more favorable, shifting this associated transition to lower energies and indicating a closing of the bandgap and semiconductor behavior. Similar pressure-induced effects on the MLCT are observed in the berkelium complexes of Bk_2_[C_6_(CO_2_)_6_]­(H_2_O)_8_·2H_2_O and [(Bk­(pmtz)_2_(H_2_O)_3_)_2_(μ-pmtz)]_2_(pmtz)_2_·6H_2_O (pmtz^–^ = 5-(pyrimidyl)­tetrazolate), where the pressure-induced red-shifting and broadening of the MLCT band is only observed for the berkelium complexes and is absent from the analogous curium and californium complexes.^54,56^ This was attributed to the accessible Bk^4+^ state (Bk^3+^/Bk^4+^ = −1.4 V vs. S.H.E), causing charge transfer to become more favorable with the application of pressure.^54,56^ Likewise, the strong shifting of the MLCT band for CsTb­(CrO_4_)_2_ could be due to the somewhat accessible Tb^4+^ state (Tb^3+^/Tb^4+^ = −3.1 V vs S.H.E).

**Figure 3:**
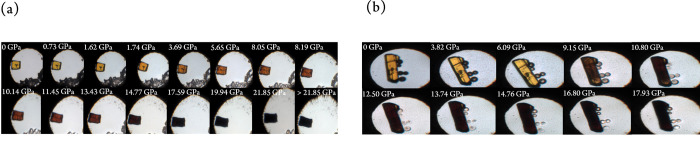
Pictures of crystals of (a) CsTb­(CrO_4_)_2_ and (b) CsDy­(CrO_4_)_2_ in a diamond anvil cell under increasing pressure.

However, one should not arrive at such conclusions with just this result. The pressure-dependent UV-vis-NIR spectra for CsDy­(CrO_4_)_2_ are presented in Figure 2b, where the lanthanide ion in this compound, Dy, is more redox-inert with a Dy^3+/4+^ oxidation potential calculated to be between −4.5 V and −5.2 V.^1^ There are a few known Dy^4+^ materials characterized in the literature, such as Cs_3_DyF_7_ that required F_2(*l*)_ at 170 bar and 693 K to synthesize.^63^ Therefore, CsDy­(CrO_4_)_2_ was still deemed to be a redox-inert analog for CsTb­(CrO_4_)_2_ due to its more largely negative oxidation potential.^1,64–66^ In CsDy­(CrO_4_)_2_, there is an extent of broadening and red-shifting of the LMCT band from the CrO_4_^2–^ despite the redox inactivity of the Dy^3+^ center. This likely does not include the lanthanide metal at all. Instead, this observation could be the result of charge transfer between neighboring chromate subunits that are brought closer together with the application of pressure. As the structure is compressed, the oxygen atoms are brought closer to a neighboring chromium metal center to the point of bridging and induce charge transfer to the neighboring chromium center. Thus, under this hypothesis, the color change in the CsDy­(CrO_4_)_2_ compound is attributed to charge transfer interactions between chromate units, similar to what is observed in common dichromate and trichromate salts, where these salts are typically a red-orange and red color, respectively. It should also be noted that the broadening of the MLCT band is reversible upon the release of pressure in both compounds (Figure S7).

To further analyze the origin of this color change for both of these lanthanide chromate compounds, high-pressure Raman spectroscopy was performed. This technique is commonplace in the general high-pressure literature because it can provide valuable information for structural changes and rearrangements that occur with the application of high pressure.^67–69^ The high-pressure Raman spectra, separated into various wavenumber regions for CsTb­(CrO_4_)_2_ and CsDy­(CrO_4_)_2_ are provided in Figure 4. The full ambient-pressure and high-pressure Raman spectra, in addition to peak assignments, can be found in the Supporting Information (Figures S8–S9 and Tables S4–S5). At ambient pressure, the observed Raman modes of the CrO_4_^2–^ units were the ν_2_ symmetric bending between 330–360 cm^–1^, the ν_4_ antisymmetric bending between 380–450 cm^–1^, ν_1_ symmetric stretching between 865–885 cm^–1^, and ν_3_ antisymmetric stretching between 825–830 cm^–1^ and 965–975 cm^–1^. There are also low intensity Ln–O vibrational modes observed between 500–700 cm^–1^ and external lattice modes below 260 cm^–1^.^70–74^

**Figure 4:**
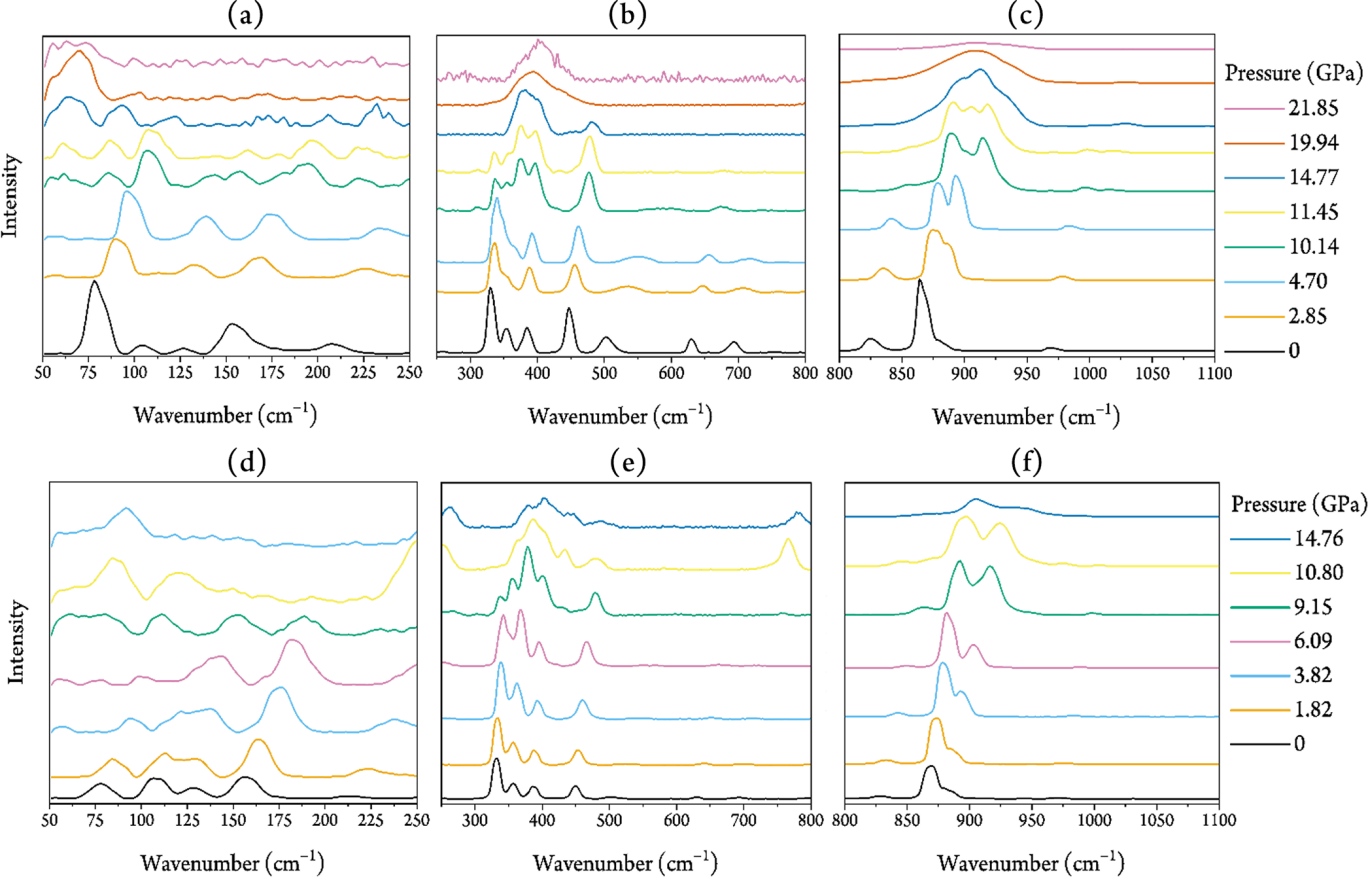
High-pressure Raman spectra of CsTb­(CrO_4_)_2_ in the regions of (a) 50–250 cm^–1^, (b) 250–800 cm^–1^, and (c) 800–1100 cm^–1^ in addition to high-pressure Raman spectra of CsDy­(CrO_4_)_2_ in the regions of (d) 50–250 cm^–1^, (e) 250–800 cm^–1^, and (f) 800–1100 cm^–1^.

Generally, the high-pressure effect on the Raman shifts in both lanthanide chromate compounds is similar, with substantial shifting to higher energy, broadening, splitting, and the diminishing of intensity at the highest pressures tested here. The shifting to higher energies observed here is due to the compression of the chemical bonds that causes these vibrational modes to occur at higher energy and is common in the literature.^67–69,75,76^ For the ν_2_ symmetric bending (330–360 cm^–1^) and ν_4_ antisymmetric bending (380–450 cm^–1^) modes in CsTb­(CrO_4_)_2_ (Figure 4b) and CsDy­(CrO_4_)_2_ (Figure 4e), similar pressure-induced effects are observed, where these signals are shifted to higher energies, broadened, decrease in intensity, and eventually substantially overlap to the point where some modes cannot be differentiated.

The difference between these two compounds can be observed in the high- and low-wavenumber regions. In the high-wavenumber region of 800–1100 cm^–1^ for CsTb­(CrO_4_)_2_ (Figure 4c), the ν_1_ symmetric stretch at 864 cm^–1^ with a shoulder at higher wavenumbers, is split to a larger degree, resulting in three distinct peaks at 11.45 GPa. At ambient pressure for CsDy­(CrO_4_)_2_, two peaks are observed at 870 cm^–1^ and 883.7 cm^–1^, but under pressure, no further splitting of these two signals is observed (Figure 4f). As for the low-wavenumber region below 250 cm^–1^ that contains the external lattice vibrations, significant changes can be observed in the high-pressure spectra of CsTb­(CrO_4_)_2_ (Figure 4a) starting at 10.14 GPa, with splittings of the peaks around 100, 140, and 185 cm^–1^ of the previous pressure step of 4.70 GPa, as well as more peaks arising below 100 cm^–1^. Comparing this to CsDy­(CrO_4_)_2_, there are less obvious changes that occur in the pressure-dependent Raman spectra in the phonon region (Figure 4d), but concrete conclusions are difficult to draw based on this data due to the overall diminished intensity of the Raman signals with the application of pressure. Despite this, with the significant changes under pressure for the CsTb­(CrO_4_)_2_ compound compared to the redox-inert CsDy­(CrO_4_)_2_ in the ν_1_ symmetric stretches and the phonon modes, there seems to be a rearrangement of the lattice that could be a sign of underlying redox chemistry occurring between the Tb^3+^ metal center and the coordinating CrO_4_^2–^ anions.

To finally determine the origin of the effects observed in the high-pressure UV–vis-NIR and Raman spectra for CsTb­(CrO_4_)_2_, high-pressure Tb^3+^ L_3_-edge high-energy-resolution fluorescence-detected X-ray absorption near-edge structure (HERFD-XANES) was performed and is provided in Figure 5. At ambient (Figure S10) and low (Figure 5) pressures, a single intense edge feature is observed around 7517 eV, consistent with Tb^3+^.^6^

**Figure 5:**
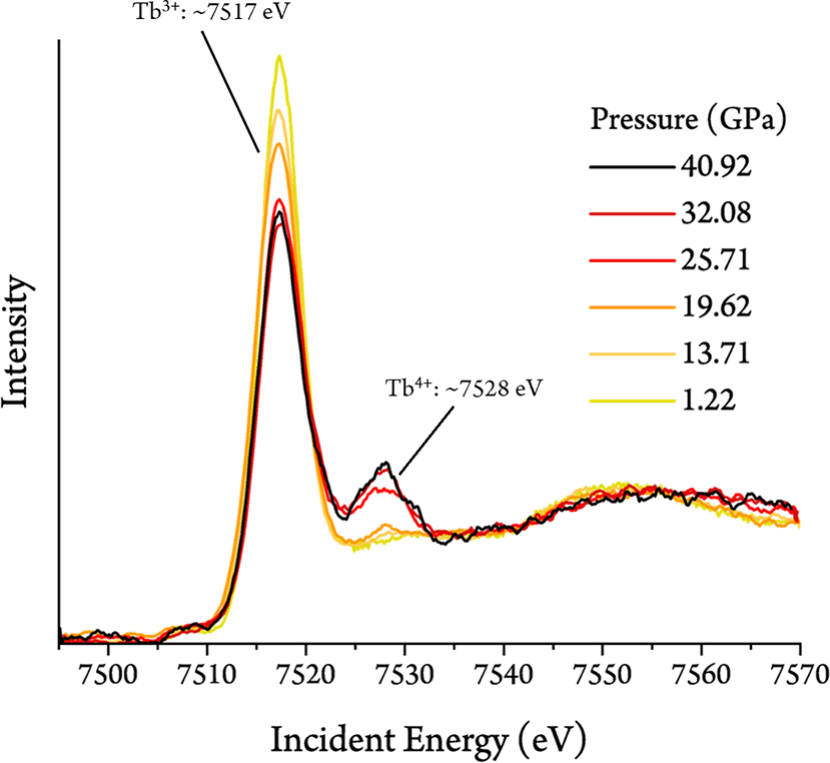
High-pressure Tb L_3_-edge HERFD-XANES spectra for CsTb­(CrO_4_)_2_.

Upon compression, the ingrowth of a second edge feature around 7528 eV is observed starting at 19.62 GPa and grows in intensity with the subsequent increase in pressure up to an experimental limit of 40.92 GPa. The appearance and growth of this peak are also correlated with a decrease in the peak intensity of the characteristic Tb^3+^ peak around 7517 eV as the pressure increases. Peak positions of the high-pressure HERFD-XANES spectra are reported in the Supporting Information (Table S6). The ingrowth of the 7528 eV peak is consistent with the existence of Tb^4+^, as indicated by previous L_3_-edge XAS spectra for Tb^4+^ complexes.^6,77–80^ For reference, recent reports of L_3_-edge XAS spectra found these characteristic Tb^4+^ double-edge features at 7518.9 and 7526.1 eV for TbO_2_ and 7520.3 and 7528.5 eV for [Tb^4+^(NP­(1,2bis-^*t*^Bu-diamidoethane)­(NEt_2_))_4_].^7,81^ Comparing these data to the Tb^3+^ L_3_-edge HERFD-XANES reported here, this L_3_-edge HERFD-XANES data provides strong evidence that tetravalent terbium is stabilized in CsTb­(CrO_4_)_2_ with the application of pressure in a DAC starting at 19.62 GPa. Furthermore, the percentage of Tb^4+^ stabilized at the reported pressure steps was quantified as 3% at 19.62 GPa, 21% at 25.71 GPa, 25% at 32.08 GPa, and 27% at 40.92 GPa. The relative concentration error was estimated to be on the order of 5%, as defined by the ITFA package. It should also be noted that the pre-edge feature around 7508 eV is unaffected by pressure up to an experimental limit of 40.92 GPa.

The contributions from the two Tb oxidation states were quantified using iterative transformation factor analysis (ITFA) and Athena software.^82^ It was found that ITFA produced higher-quality peak fittings than Athena, so ITFA was chosen for the quantification provided here (Figure S12). The ITFA procedure was applied to the normalized and as-measured Tb L_3_ HERFD-XANES spectra. The analysis was carried out in several steps. First, principal component analysis (PCA) was performed to determine the number of components contributing to the HERFD-XANES spectra. The PCA results indicated that the spectra are well reproduced by a linear combination of two components, corresponding to Tb^3+^ and Tb^4+^, while the third, fourth, fifth, sixth, and seventh components show no significant contributions. In the second step, CsTb­(CrO_4_)_2_ at 1.22 GPa and TbO_2_ under ambient conditions were used as standards and assumed to contain 100% Tb^3+^ and 100% Tb^4+^, respectively. The iterative target test (ITT) procedure was then applied to determine the relative concentrations of the two components as reported above. The relative concentration error was estimated to be on the order of 5%, as defined by the ITFA package.

It should be noted that with the partial oxidation of Tb^3+^, there must also be some chemical species that are reduced. Owing to the rich redox chemistry of Cr, it is likely that Cr^6+^ is partially reduced to Cr^5+^, as Tb^4+^ is stabilized under pressure. To evaluate this hypothesis, Cr L_3_-edge HERFD-XANES was performed on CsTb­(CrO_4_)_2_ inside the DAC; however, Cr^6+^ impurities were found in the beryllium gasket alloy that houses the sample. This caused the signal of the single crystal of CsTb­(CrO_4_)_2_ to be heavily overshadowed by the signal produced from the Cr^6+^ in the beryllium gasket, and the partial reduction of Cr^6+^ was unable to be experimentally confirmed by these methods. Nonetheless, the appearance of double-edge features at 7517.49 and 7528 eV in the high-pressure Tb L_3_-edge HERFD-XANES provided sufficient evidence that the Tb^4+^ is stabilized under high pressure starting at 19.62 GPa, and this is likely associated with the concurrent partial reduction of Cr^6+^ to Cr^5+^.

## Conclusions

The high-pressure Tb L_3_-edge HERFD-XANES, supplemented with high-pressure UV-vis-NIR and Raman spectroscopy reported here, supports the stabilization of Tb^4+^ in CsTb­(CrO_4_)_2_ starting at 20 GPa and increasing the Tb^4+^ content with additional pressure. This represents the first example of pressure-induced oxidation of Tb to stabilize the tetravalent state and thus provides an alternative pathway for accessing the tetravalent state without the need for an inert atmosphere or extreme oxidants. These results suggest that accessing uncommon high-valent oxidation states in the *f* elements may be more achievable than previously perceived by applying high pressure.

The pressure that Tb^4+^ is first observed is associated with an almost completely diminished signal in the Raman spectrum and a dark red/black color of the crystal, stemming from the red shifting of the MLCT band as indicated by the high-pressure UV-vis-NIR spectra. Comparing CsTb­(CrO_4_)_2_ to CsDy­(CrO_4_)_2_ with the more redox-inert dysprosium lanthanide ion, the high-pressure Raman and UV-vis-NIR spectra yield similar results. Previous reports have attributed pressure-induced red shifting of an MLCT band to other accessible oxidation states in the lanthanides and actinides, but the similar results presented here for CsDy­(CrO_4_)_2_ suggest that one must be careful when making this conclusion. The red shifting of the broad LMCT band for CsDy­(CrO_4_)_2_ could instead be due to charge-transfer effects between nearby CrO_4_^2–^ units that are amplified under pressure.

## Supplementary Material



## Notes

Images of products; synthesis yields; thermal ellipsoid plots; powder X-ray diffraction patterns; crystallographic data; ambient pressure UV-vis-NIR, and Raman spectra; UV-vis-NIR spectra after the release of pressure; high-pressure Raman spectra across the entire spectral range; peak assignments for Raman spectra; high pressure; and HERFD-XANES peak positions (PDF)
