# Supplementary material for: Pressure-Induced Stabilization of Terbium(IV) in CsTb(CrO4)2 Characterized by X‑ray Absorption Spectroscopy
Source: J Am Chem Soc. 2026 Jun 16;148(25):26555–63. doi: 10.1021/jacs.6c06234 (PMC13339621; doi:10.1021/jacs.6c06234)
Supplement: Supplementary file 1 [file ja6c06234_si_001.pdf]

Supporting Information for

Pressure-Induced Stabilization of Terbium(IV) in $CsTb(CrO_4)_2$ Characterized by X-ray Absorption Spectroscopy

Tyler W. Hines[a], Lucia Amidani[b,c], Nicholas B. Beck[a], Kacy N. Mendoza[a], Christoph Sahle[d], Joseph M. Sperling[a], Sami Vasala[b,c], Kristina O. Kvashnina[b,c]*, Thomas E. Albrecht[a]*

[a]Department of Chemistry and Nuclear Science & Engineering Center, Colorado School of Mines, Golden, Colorado 80401, United States

[b]The Rossendorf Beamline, European Synchrotron Radiation Facility, 71, Avenue des Martyrs, CS 40220, 38043 Grenoble Cedex 9, France

[c]Helmholtz-Zentrum Dresden-Rossendorf, Institute of Resource Ecology, Bautzner Landstraße 400, 01328 Dresden, Germany

[d]European Synchrotron Radiation Facility, 71 Avenue des Martyrs, 38043 Grenoble, France

*Corresponding Authors' Emails: kristina.kvashnina@esrf.fr thomas.albrecht@mines.edu

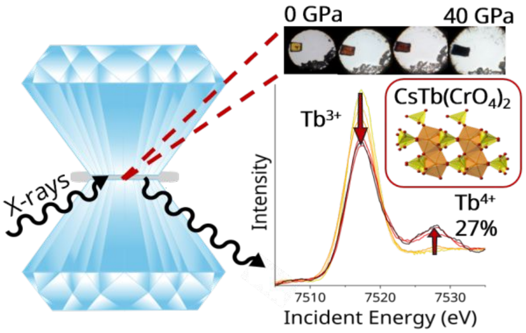

Table of Contents

Product Photographs S3

Synthetic Amounts and Yields S3

Thermal Ellipsoid Plots of Asymmetric Units S3

Powder X-ray Diffraction Patterns S4

Crystallographic Information S5

Ambient Pressure UV-vis-NIR spectra S7

High Pressure UV-Vis-NIR spectra of $CsDy(CrO_4)_2$ from 700 to 1200 nm S7

Ambient Pressure Raman Spectra S8

Full High Pressure Raman Spectra S9

High Pressure Raman Spectra Peak Assignments S10

Ambient Pressure Tb^{3+} HERFD-XANES Spectrum S12

High Pressure Tb^{3+} HERFD-XANES Peak Positions S12

ITFA HERFD-XANES Curve Fittings S13

Athena HERFD-XANES Curve Fittings S13

(a) 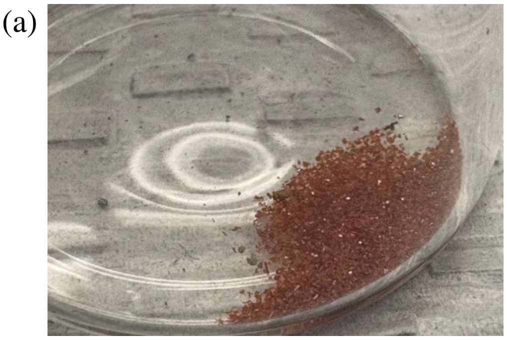 (b) 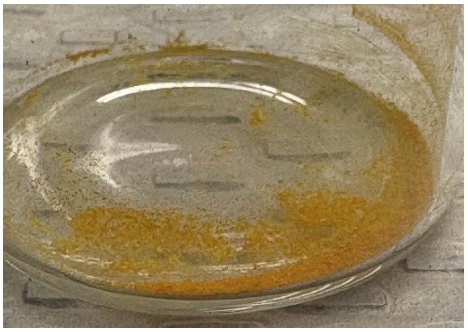

Figure S1. Pictures of the bulk crystalline products of (a) $CsTb(CrO_4)_2$ and (b) $CsDy(CrO_4)_2$.

Table S1. Masses for Syntheses and Respective Yields

Compound	Cs_2CrO_4 Mass	$Ln(NO_3)_3 \cdot 6H_2O$ Mass	Product mass	Percent Yield
$CsTb(CrO_4)_2$ - 1	153.4 mg	92.7 mg	82.4 mg	89.2%
$CsTb(CrO_4)_2$ - 2	153.8 mg	90.5 mg	56.7 mg	61.7%
$CsTb(CrO_4)_2$ - 3	152.4 mg	92.7 mg	49.6 mg	54.0%
$CsDy(CrO_4)_2$ - 1	167.2 mg	103.2 mg	64.7 mg	63.8%
$CsDy(CrO_4)_2$ - 2	153.3 mg	91.4 mg	73.8 mg	79.6%
$CsDy(CrO_4)_2$ - 3	154.2 mg	92.1 mg	68.5 mg	75.5%

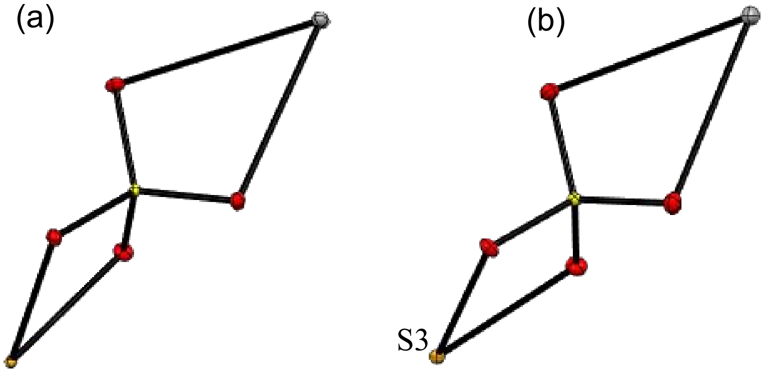

Figure S2. Thermal ellipsoid plots drawn at 50% probability for the asymmetric unit of (a)

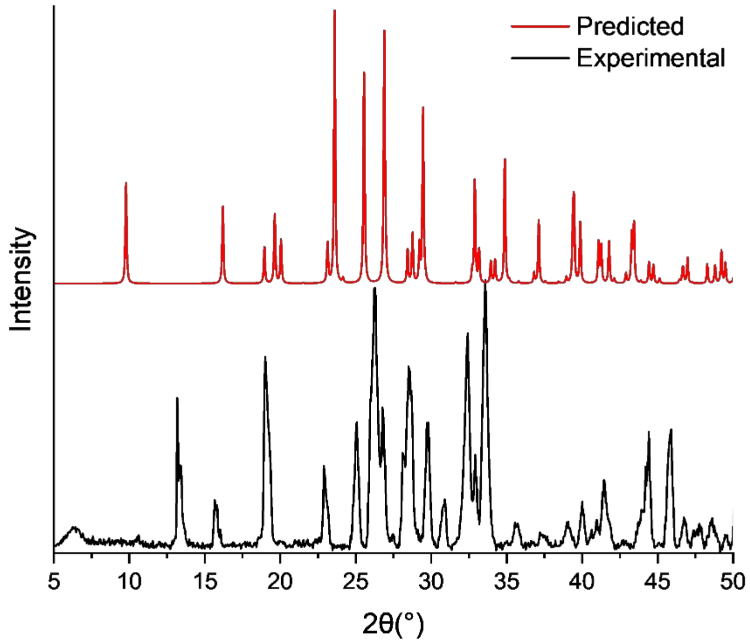

Figure S3. Experimental (a) and calculated (b) powder patterns for $CsTb(CrO_4)_2$.

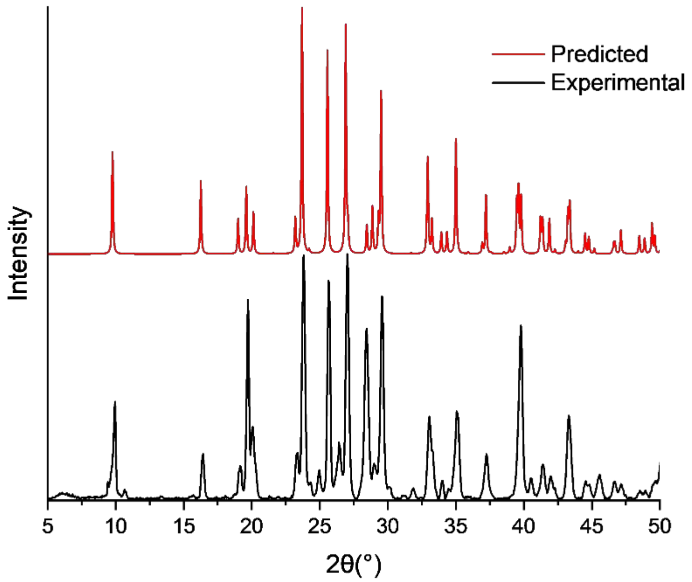

Figure S4. Experimental (a) and calculated (b) powder patterns for $CsDy(CrO_4)_2$.

Table S2. $CsTb(CrO_4)_2$ Crystallographic data

Temperature/K	104.50
Crystal System	monoclinic
Space Group	$P2/c$
a/Å	9.1409(6)
b/Å	5.4702(4)
c/Å	7.6208(6)
β/°	98.750(3)
Volume/Å^3	376.62(5)
Z	2
ρcalcg/cm^3	4.619
μ/mm^{-1}	16.887
F(000)	464.0
Crystal size/mm^3	0.186 × 0.101 × 0.085
Radiation	Mo K_α (λ = 0.71073 Å)
2Θ range for data collection/°	4.508 to 61.212
Index ranges	$-13 \leq h \leq 13$, $-7 \leq k \leq 7$, $-10 \leq l \leq 10$
Reflections collected	7415

Independent reflections	1112 [R_{int} = 0.0306, R_{sigma} = 0.0201]
Data/restraints/parameters	1112/0/57
Goodness-of-fit on F^2	1.152
Final R indexes [I≥2σ (I)]	R_1 = 0.0131, wR_2 = 0.0308
Final R indexes [all data]	R_1 = 0.0132, wR_2 = 0.0309
Largest diff. peak/hole / e Å^{-3}	0.57/−0.86

Table S3. $CsDy(CrO_4)_2$ Crystallographic data

Temperature/K	150
Crystal System	monoclinic
Space Group	*P*2/*c*
a/Å	9.1609(5)
b/Å	5.4493(3)
c/Å	7.5943(4)
β/°	98.826(2)
Volume/Å^3	374.62(4)
Z	4
ρcalcg/cm^3	4.676
μ/mm^{-1}	17.511
F(000)	466.0

Crystal size/mm^3	0.05 × 0.01 × 0.01
Radiation	Mo K_α (λ = 0.71073 Å)
2Θ range for data collection/°	7.478 to 61.162
Index ranges	−13 ≤ *h* ≤ 13, −7 ≤ *k* ≤ 7, −10 ≤ *l* ≤ 10
Reflections collected	23029
Independent reflections	1148 [R_{int} = 0.0413, R_{sigma} = 0.0185]
Data/restraints/parameters	1148/0/57
Goodness-of-fit on F^2	1.278
Final R indexes [I≥2σ (I)]	R_1 = 0.0120, wR_2 = 0.0320
Final R indexes [all data]	R_1 = 0.0121, wR_2 = 0.0320
Largest diff. peak/hole / e $Å^{-3}$	1.06/−0.73

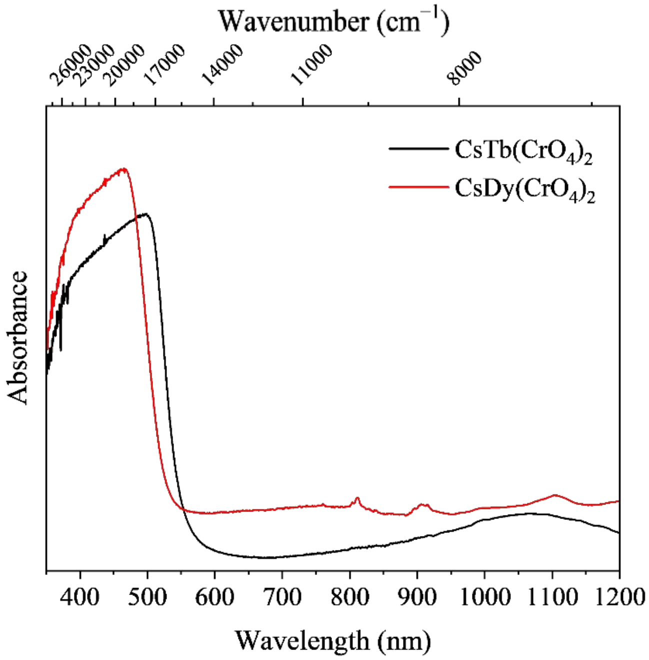

Figure S5. Ambient pressure UV-vis-NIR spectra of $CsTb(CrO_4)_2$ (black) and $CsDy(CrO_4)_2$ (red).

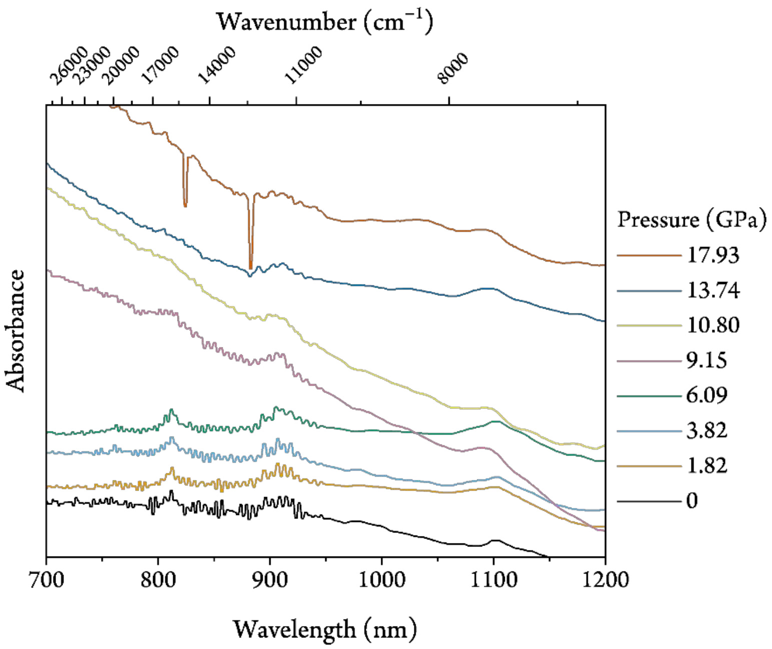

Figure S6. High pressure UV-vis-NIR spectra of $CsDy(CrO_4)_2$ zoomed in around the $f \rightarrow f$ transitions.

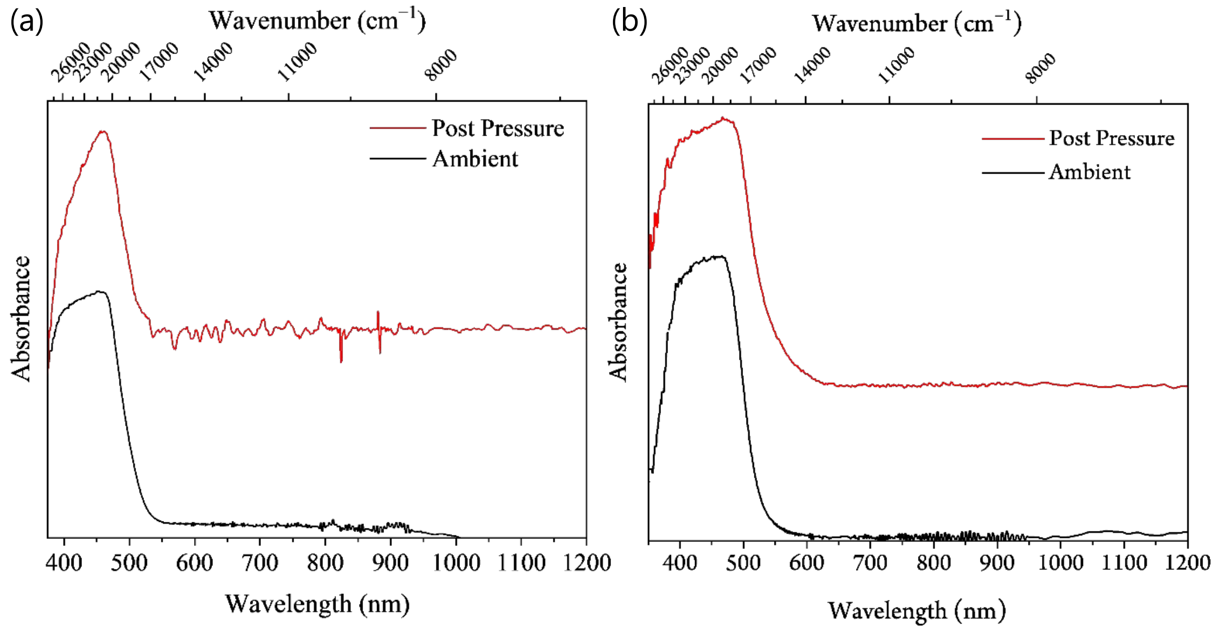

Figure S7. UV-vis-NIR spectra at ambient pressure and after the release of the pressure of (a) $CsTb(CrO_4)_2$ and (b) $CsDy(CrO_4)_2$.

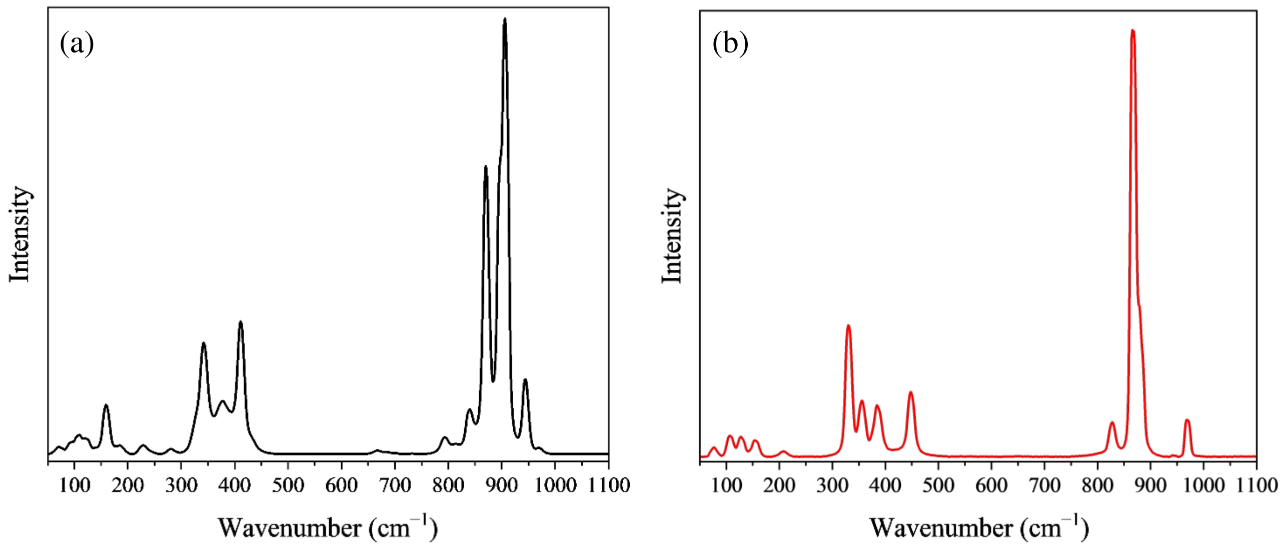

Figure S8. Ambient pressure Raman spectra of (a) $CsTb(CrO_4)_2$ and (b) $CsDy(CrO_4)_2$.

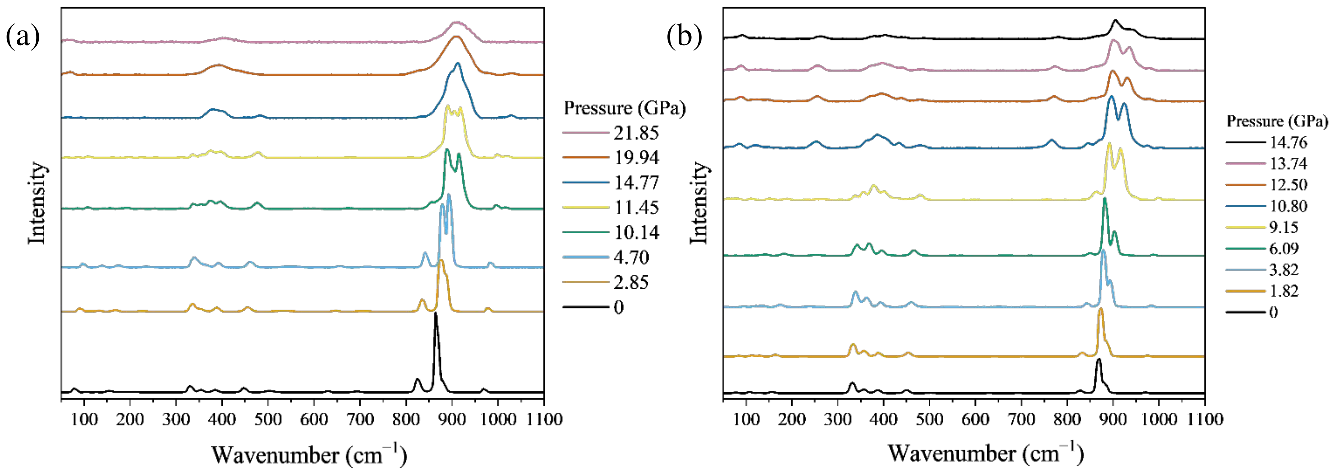

Figure S9. High pressure Raman spectra from 50 cm^{-1} to 1100 cm^{-1} for (a) $CsTb(CrO_4)_2$ and (b) $CsDy(CrO_4)_2$.

Table S4. High pressure Raman assignments for $CsTb(CrO_4)_2$. Color coded rows are to identify peaks that shift with the application of pressure and the average shift (cm^{-1}/GPa) column denotes the average shift of these respective peaks.

Assignment	0 GPa (cm^{-1})	2.85 GPa (cm^{-1})	4.7 GPa (cm^{-1})	10.14 GPa (cm^{-1})	11.45 GPa (cm^{-1})	14.77 GPa (cm^{-1})	19.94 GPa (cm^{-1})	21.85 GPa (cm^{-1})	Average Shift (cm^{-1}/GPa)
Internal Lattice Modes		57.1	54	55.5	61.5	64.5	70.5	65.6	
			60	61.5	67.2	71.7	98.1		
			76.4	68.0	87	93	102.7		
				85.5					
	78	90	96	106.5	109.5	121.5			2.945
	105	112.5		144	162	168	167.6		3.139
	127.2	132	139.5	157.5	178.5	172.5	172.7		3.676
			172.5	181.5		181.5			
	153	169.5	176.9	195	198	205.5			3.555
	177.2			222	222	232.5			
					226.5				
					232.6				
					252				
	207	225	232.5	253.5	259.5				4.585
			251.6	274.3	275.0				3.461
	257.1			281.4					2.405
					312				
				309	318.0				6.871
				337.5	336				
ν_2	330	336	339	354	359.0123				2.534
	354	354.5	365.3	375	376.5	382.5			1.930
ν_4	384	388.5	391.5	397.5	399	400.5	394.5	404.50	1.117
					433.5	448.5			
						457.5			
	447	456	460.5	477	478.5	481.5			2.336
Ln−O	502.5	537	550.5						10.213
	630	648	655.5	673.5	679.0				4.275
	693	709.5	717.0						5.116
ν_3				806.2	811.1	832.8	831.0		2.523
	825	835.5	841.5	856.5					3.107
ν_1	864	874.5	879	889.5	891	898.4			2.330
		888.2	892.5	903	906	913.5			2.122
ν_3				915	918		910.5	911.5	
					970.2	972.7	980.5		
	967.5	976.5	982.5	996	997.5				2.620
						1003.6			
				1017	1020	1028.6	1030.3		

Table S5. High pressure Raman assignments for $CsDy(CrO_4)_2$. Color coded rows are to identify peaks that shift with the application of pressure and the average shift (cm^{-1}/GPa) column denotes the average shift of these respective peaks.

**Assignment**	**0 GPa (cm^{-1})**	**1.82 GPa (cm^{-1})**	**3.82 GPa (cm^{-1})**	**6.09 GPa (cm^{-1})**	**9.15 GPa (cm^{-1})**	**10.8 GPa (cm^{-1})**	**12.5 GPa (cm^{-1})**	**13.74 GPa (cm^{-1})**	**14.76 GPa (cm^{-1})**	**Average Shift (cm^{-1}/GPa)**
Internal Lattice Modes			57	55.5	61.5	66	88.5	88.5	91.5	
				78	69	84		253.5		
					79.5					
	78	87	94.5	99	111	120				3.889
	106.5	114	121.5							3.927
	127.5	135	138	144	153					2.787
	156	163.5	175.5	181.5	189					3.607
					232.0					
					253.5					
	211.5	225	238.5	252	264					5.738
					303.1					
						252	256.5	258	264	3.030
ν_2					337.5					
	331.5	333	339	342	355.5	363.9	376.0		379.5	3.252
	357	357	361.5	367.5	378	387	394.5	399	402	3.049
ν_4	385.5	387	391.5	394.5	400.5	406.5				
									438	
					429	433.5	438	438	447	3.209
					456.5					
	450	453	460.5	466.5	480	481.5	481.7	481.7	486.4	2.466
Ln−O	502.5	523.5								11.538
	630	640.5	649.5							3.202
	693	697.5								5.769
		703.5								
					756.4	766.5	771	774.1	780.29	4.254
ν_3						844.5	855	865.4	869.5	6.321
	828	832.5	841.5	849	864	870.6				3.942
ν_1	870	874.5	879	882	892.5	897	900	900	904.5	2.337
	883.7	886.1	892.5	903	916.5	924	930	936	937.5	3.645
ν_3						975	978	980.6	986.8	2.977
	972	973.5	982.5	987	999.2	1005.5				3.099
					1027.3					

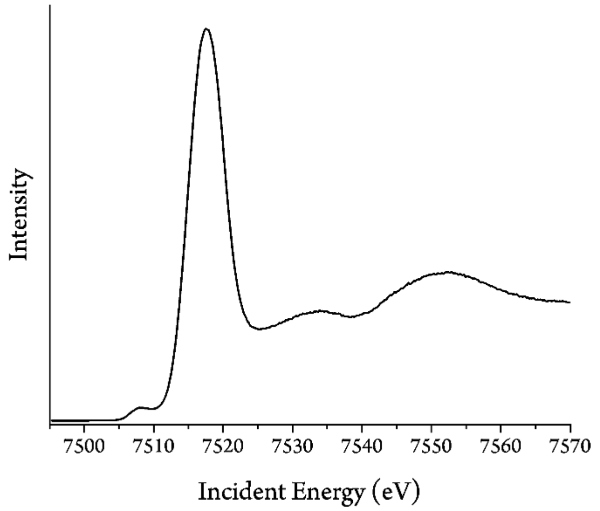

Figure S10. Ambient pressure Tb^{3+} L_3-edge HERFD-XANES spectra for $CsTb(CrO_4)_2$

Table S6. High Pressure Tb^{3+} L_3-edge HERFD-XANES Peak Positions

Pressure (GPa)	Tb^{3+} Peak Position (eV)	Tb^{4+} Peak Position (eV)
Ambient	7517.78	N/A
1.22	7517.56	N/A
13.71	7517.36	N/A
19.62	7517.22	7528.21
25.71	7517.53	7527.22
32.08	7517.60	7527.01
40.92	7517.40	7526.90

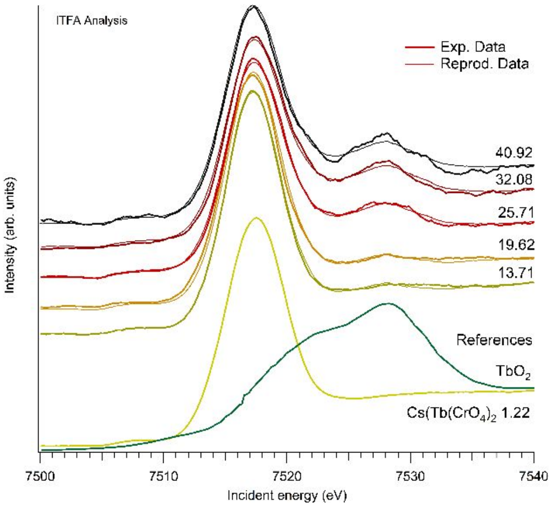

Figure S11. Curve fittings compared to the high pressure L_3-edge HERFD-XANES experimental data using the ITFA software.

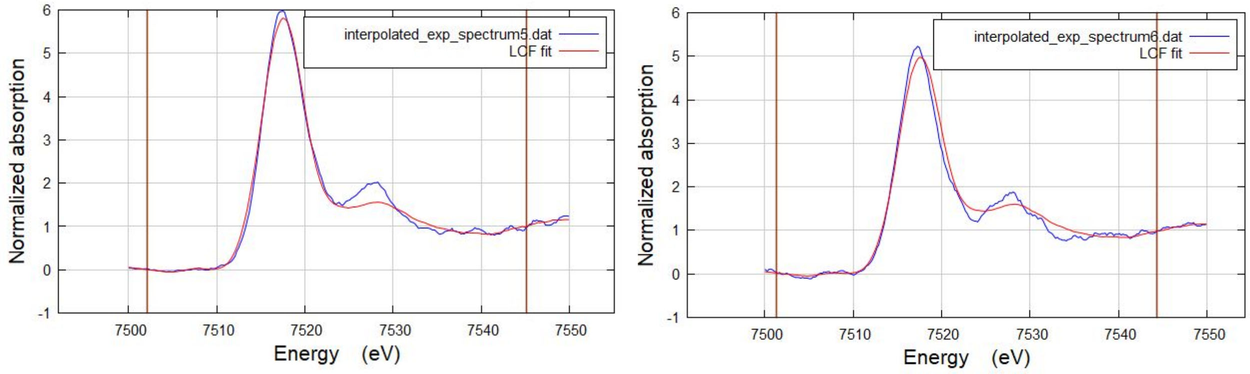

Figure S12. Curve fittings compared to the high pressure L_3-edge HERFD-XANES experimental data using the Athena software.
